# Redundancy in the World of MAP Kinases: All for One

**DOI:** 10.3389/fcell.2016.00067

**Published:** 2016-06-27

**Authors:** Marc K. Saba-El-Leil, Christophe Frémin, Sylvain Meloche

**Affiliations:** ^1^Institute for Research in Immunology and Cancer, Université de MontréalMontréal, QC, Canada; ^2^Institute for Research in Cancer of MontpellierMontpellier, France; ^3^Molecular Biology Program, Université de MontréalMontréal, QC, Canada; ^4^Department of Pharmacology, Université de MontréalMontréal, QC, Canada

**Keywords:** signal transduction, MAP kinases, ERK1/2, JNK, p38, mouse genetics, functional redundancy

## Abstract

The protein kinases ERK1 and ERK2 are the effector components of the prototypical ERK1/2 mitogen-activated protein (MAP) kinase pathway. This signaling pathway regulates cell proliferation, differentiation and survival, and is essential for embryonic development and cellular homeostasis. ERK1 and ERK2 homologs share similar biochemical properties but whether they exert specific physiological functions or act redundantly has been a matter of controversy. However, recent studies now provide compelling evidence in support of functionally redundant roles of ERK1 and ERK2 in embryonic development and physiology. In this review, we present a critical assessment of the evidence for the functional specificity or redundancy of MAP kinase isoforms. We focus on the ERK1/ERK2 pathway but also discuss the case of JNK and p38 isoforms.

## Introduction

Mitogen-activated protein (MAP) kinase pathways are evolutionarily conserved signaling modules that play a key role in transducing extracellular signals into intracellular responses (Meloche, 2012). These signaling modules are found in plants, fungi and animals (Kultz, 1998). In mammals, 14 MAP kinase genes have been identified that define 7 distinct MAP kinase pathways. The best-characterized MAP kinase pathways are the extracellular signal-regulated kinase 1(ERK1)/2, cJun NH_2_-terminal kinase 1 (JNK1)/2/3, and p38α/β/γ/δ pathways. Phylogenetic analysis of the evolutionary history of MAP kinase genes suggests that vertebrate MAP kinases originated from 3 precursors and have expanded through gene duplication during early vertebrate evolution (Li et al., 2011). Thus, invertebrate species have less MAP kinases than vertebrate species. For example, humans express two ERK isoforms, ERK1 and ERK2, whereas Drosophila expresses the single ortholog Rolled. The expansion of vertebrate MAP kinase genes raises the important question of whether mammalian MAP kinase isoforms have evolved unique physiological functions or are used interchangeably to reach a threshold of global kinase activity. The current review addresses this question.

## ERK1 and ERK2, two homologous kinases with similar biochemical properties

ERK1 and ERK2 are the effector kinases of the prototypical Ras-ERK1/2 MAP kinase pathway. This signaling pathway processes information from a wide range of extracellular stimuli to regulate cell proliferation, differentiation and survival (Pearson et al., 2001). ERK1 and ERK2 isoforms are encoded by distinct genes, which are located on chromosomes 16q11 and 22q11 in human, respectively (Li et al., 1994). They are co-expressed in almost all cell types and tissues, although their relative abundance varies considerably from one tissue to another (Boulton and Cobb, 1991; Boulton et al., 1991; Fremin et al., 2015). Of note, some discrete regions of the adult mouse brain express exclusively *Erk1* or *Erk2* mRNA, suggesting that a single ERK isoform mediates cellular responses in these areas (Di Benedetto et al., 2007). The two ERK proteins display 83% amino acid identity overall and 100% similarity in residues involved in catalysis and docking interactions with substrates (Boulton et al., 1990, 1991; Busca et al., 2015). They share similar biochemical properties and are activated by the upstream kinases MEK1/2 with comparable efficiency *in vitro* (Robbins et al., 1993). Hydrogen/deuterium exchange mass spectrometry has revealed distinct patterns of activation-induced changes in conformational mobility between ERK1 and ERK2 (Ring et al., 2011). However, these differences in internal protein motions do not appear to significantly impact protein kinase activity and selectivity. ERK1 and ERK2 both recognize the same minimal Ser/Thr-Pro primary sequence determinant on substrates, with a preference for a proline at P-2 position (Gonzalez et al., 1991), and have almost identical *in vitro* intrinsic kinase activity (Lefloch et al., 2008). They phosphorylate hundreds of substrates (Yoon and Seger, 2006; Courcelles et al., 2013) and, with the exception of a few anecdotal reports (Chuang and Ng, 1994; Hanlon et al., 2001; Hwang et al., 2009), no evidence has been provided for a difference in substrate specificity between the two isoforms. Quantitative proteomics analysis of the ERK1 interactome in agonist-stimulated PC12 cells led to the identification of 284 ERK1-interacting proteins (von Kriegsheim et al., 2009). Notably, all proteins tested also interacted with ERK2 in co-immunoprecipitation assays. Thus, the two ERK isoforms display similar biochemical properties.

## Evidence for specific regulatory mechanisms and functions of ERK1 and ERK2

The question of whether ERK1 and ERK2 exerts specific functions or act redundantly has been a subject of intense research and controversy over the years. The unavailability of activated alleles or selective pharmacological inhibitors of ERK1 and ERK2 has complicated the analysis of their functions. Expression of phosphorylation-defective or catalytically inactive mutants of ERK1 or ERK2 has been used to successfully probe the functions of the kinases, but these mutants exert dominant interfering effects on both isoforms (Pages et al., 1993). The development of small molecule pharmacological inhibitors of MEK1/2, such as PD98059 and U0126, has provided invaluable tools for dissecting out the role of the ERK1/2 MAP kinase pathway in numerous cellular responses (Dudley et al., 1995; Favata et al., 1998; Fremin and Meloche, 2010). However, these reagents could not be used to discriminate the roles of each isoform.

A number of studies have reported that ERK1 and ERK2 are regulated differentially in response to specific extracellular stimuli or cellular contexts (Papkoff et al., 1994; English and Sweatt, 1996; Kashiwada et al., 1996; Sarbassov et al., 1997; Matos et al., 2005; Wollmann et al., 2005; Aceves-Luquero et al., 2009; Chernova et al., 2009). However, these results should be interpreted with caution as they rest on the use of non-quantitative immunoblotting assays to monitor the activating phosphorylation of ERK isoforms. In the vast majority of studies, ERK1 and ERK2 were found to be co-activated in response to various extracellular agonists (Lewis et al., 1998). Detailed kinetic analyses in mouse fibroblasts have revealed that the two ERK isoforms are coordinately phosphorylated and enzymatically activated in response to mitogenic factors (Meloche, 1995). Intriguingly, the scaffold protein MP1 (MEK Partner 1) was proposed to interact preferentially with MEK1 and ERK1, and to specifically enhance ERK1 activation (Schaeffer et al., 1998). MP1 was later shown to form a heterodimeric complex with the adaptor protein p14, which is required to localize MP1 to late endosomes and promote the endosomal activation of both ERK1 and ERK2 isoforms (Wunderlich et al., 2001; Teis et al., 2002). Conditional deletion of the *p14* gene in the mouse epidermis further demonstrated that p14 is required for the global activation of ERK1 and ERK2 in the epidermis (Teis et al., 2006), refuting the isoform-specific regulatory function of the MP1-p14 scaffolding complex. It has also been reported that the nucleocytoplasmic trafficking of ERK1 is slower than that of ERK2 because of a unique sequence located in the N-terminal extremity of ERK1 (Marchi et al., 2008, 2010). As a consequence, ERK1 would have a reduced capability of transducing proliferative signals to the nucleus. However, this model requires rigorous validation by other groups and is inconsistent with genetic studies of the individual roles of ERK1 and ERK2 in cell proliferation (see below).

The generation of genetically-engineered mouse models bearing inactivating mutations in the *Erk1* and *Erk2* genes and the advent of RNA interference (RNAi) technology has allowed analysis of the phenotypical consequences of the specific depletion of ERK1 or ERK2 in animals and cells. *Erk1*^−∕−^ mice develop normally, are viable and fertile, and display no observable phenotype (Pages et al., 1999). In contrast, invalidation of the *Erk2* gene in mouse severely compromises the formation of ectoplacental cone and extra-embryonic ectoderm, which give rise to mature trophoblast derivatives in the fetus (Saba-El-Leil et al., 2003). Therefore, *Erk2* disruption leads to embryonic lethality early in mouse development after the implantation stage at embryonic day (E) 6.5 (Hatano et al., 2003; Saba-El-Leil et al., 2003; Yao et al., 2003). These observations suggested for the first time that ERK1 and ERK2 could exert specific biological functions *in vivo*. Further analysis of ERK1- or ERK2-deficient mice has fueled the idea of isoform-specific functions. Thus, it has been proposed that ERK1 specifically regulates adipocyte differentiation (Bost et al., 2005), skin homeostasis and carcinogenesis (Bourcier et al., 2006), cocaine-sensitive long-term depression of excitatory synaptic transmission (Grueter et al., 2006), splenic erythropoiesis (Guihard et al., 2010), and osteoclast differentiation (He et al., 2011; Saulnier et al., 2012). A defect in thymocyte maturation was originally described in ERK1-deficient mice (Pages et al., 1999) but subsequent studies failed to confirm this phenotype (Fischer et al., 2005; Nekrasova et al., 2005). Contradictory findings have also been reported about the specific role of ERK1 in emotional learning and memory (Selcher et al., 2001; Mazzucchelli et al., 2002). Heterozygous inactivation and conditional deletion of *Erk2* have been used to study the role of the kinase in specific tissues. These studies have suggested that ERK2 preferentially regulates cardiac myocytes survival (Lips et al., 2004), CD8 T cell proliferation and survival (D'Souza et al., 2008), neural development and associated cognitive functions and memory (Satoh et al., 2007; Samuels et al., 2008), production of brain collagen (Heffron et al., 2009), nociceptive sensitization (Alter et al., 2010), oligodendrocyte differentiation (Fyffe-Maricich et al., 2011), and social behaviors (Satoh et al., 2011a).

*In vitro* studies of cells depleted of ERK1 or ERK2 expression by genetic disruption or RNAi have also contributed to the idea of isoform-specific functions. For example, ERK1-deficient keratinocytes show an impaired proliferative response to mitogenic factors (Bourcier et al., 2006). On the other hand, depletion of ERK2 was reported to specifically impair terminal differentiation of skeletal myoblasts (Li and Johnson, 2006), replication of hepatocytes (Fremin et al., 2007; Bessard et al., 2008), transforming growth factor-beta-induced collagen synthesis (Li et al., 2009), Ras-dependent epithelial-to-mesenchymal transition (Shin et al., 2010), hepatocyte growth factor-induced lung cancer cell migration (Radtke et al., 2013), oncogenic Ras-induced senescence (Shin et al., 2013), and regulation of gp130 expression (Bonito et al., 2014). However, it should be emphasized that several other studies have documented that both ERK isoforms similarly contribute to the cellular response being studied (Liu et al., 2004; Wille et al., 2007; Lefloch et al., 2008; Dumesic et al., 2009; Voisin et al., 2010; Wei et al., 2010). Intriguingly, one group even proposed that ERK1 and ERK2 exert antagonistic effects on cell proliferation (Vantaggiato et al., 2006). This model was disproved in subsequent studies (Lefloch et al., 2008; Voisin et al., 2010).

The ERK1 gene has been reported to undergo alternative splicing to encode the Erk1b transcript in the rat (Yung et al., 2000) and ERK1c transcript in human (Aebersold et al., 2004). A subsequent study proposed that ERK1c specifically regulates Golgi fragmentation during mitosis in a non-redundant manner with ERK1 and ERK2 (Shaul and Seger, 2006). Analysis of ERK1 nucleotide sequences indicates that the Erk1b and ERK1c transcripts derive from the retention of an intronic sequence between exon 7 and exon 8 of the gene. Notably, the mouse *Erk1* gene contains an intron of 79 nucleotides at this position, but no evidence has been reported that this intron sequence can be translated in mouse tissues. The lack of an ERK1b isoform in the mouse raises doubts about the physiological importance of this isoform.

## Functional redundancy of ERK1 and ERK2: Lessons from genetic studies

The observation that specific ablation of ERK1 or ERK2 causes distinct phenotypes in cells or mice has been interpreted by many authors as evidence for isoform-specific functions of the two kinases. However, these studies did not take into account the global level of ERK1/2 activity in the analysis of the phenotypes. This is a crucial point since ERK1 and ERK2 are expressed at different levels in cell lines and tissues, with ERK2 being the predominant isoform in most tissues. Accordingly, this may explain why depletion of ERK2 usually results in stronger phenotypes than the loss of ERK1. The impact of the total activity of ERK1 and ERK2 on phenotypic outcomes was analyzed quantitatively in three *in vitro* studies. In a first study, Wille et al. generated an epi-allelic series of stable ERK1 and ERK2 knockdown mouse T cell lines obtained by shRNA lentiviral infections (Wille et al., 2007). They showed that T-cell receptor-stimulated interleukin-2 production was dependent on both total and phosphorylated ERK levels, with a similar contribution of ERK1 and ERK2. In another study, Lefloch et al. have used RNAi to silence the expression of ERK1 and ERK2 in NIH 3T3 fibroblasts and examine their relative roles in cell proliferation and immediate-early gene expression (Lefloch et al., 2008). Depletion of ERK2 slowed down the proliferation of NIH 3T3 cells, whereas reduction of ERK1 expression had no effect. Interestingly, by clamping the expression of ERK2 to a limiting level, they showed that depletion of ERK1 further restrains cell proliferation, demonstrating that both isoforms positively contributes to cell proliferation. Silencing of either ERK1 or ERK2 expression was sufficient to inhibit the serum-dependent transcriptional induction of immediate-early genes in this model. Importantly, these authors established that ERK1 and ERK2 have similar intrinsic kinase activity and demonstrated that the relative expression level of the two ERK proteins correlates with their ratio of activation state. Our group used a robust genetic approach to analyze the individual roles of ERK1 and ERK2 in cell proliferation using primary mouse embryonic fibroblasts (MEFs) as model (Voisin et al., 2010). We showed that individual loss of either ERK1 or ERK2 decreases the proliferation rate of MEFs. The impact of ERK2 deficiency was more severe, consistent with its higher level of expression in these cells. Genetic disruption of both *Erk1* and *Erk2* genes resulted in complete G1 arrest and premature replicative senescence. By combining genetic disruption of *Erk1* or *Erk2* with RNAi depletion of the alternate isoform, we were able to demonstrate that the rate of MEF proliferation is strongly correlated with the global level of phosphorylated ERK1/ERK2, which is dictated by the relative expression of the two isoforms. Altogether, these findings provided strong evidence for a redundant role of ERK1 and ERK2 in promoting cell proliferation.

*In vivo* analyses of genetically-engineered mutant mice also suggested that ERK1 and ERK2 have redundant functions in specific tissues. Conditional inactivation of *Erk2* in the developing neural crest leads to craniofacial abnormalities and conotruncal cardiac defects, which are exacerbated by the additional deletion of *Erk1* (Newbern et al., 2008). Similarly, *Erk1* deficiency enhances the abnormal neurogenesis phenotype in central nervous system-specific *Erk2* conditional knockout mice (Satoh et al., 2011b). Genetic deletion of *Erk1* and *Erk2* genes in hematopoietic cells coupled to reconstitution studies with catalytically active or inactive ERK1 and ERK2 also revealed that ERK1 and ERK2 play redundant kinase-dependent functions in the maintenance of hematopoietic stem cells and adult hematopoiesis (Chan et al., 2013; Staser et al., 2013).

We have used complementary genetic approaches to rigorously address the question of ERK1 and ERK2 specificity or redundancy in embryonic development (Fremin et al., 2015). In a first approach, we examined the impact of the progressive deletion of *Erk1* and *Erk2* alleles on the development of the placenta and embryo *per se*. We found that the weight of the placenta and surface of the labyrinth is strictly correlated with the total activity of ERK1/2 as monitored by anti-phospho-ERK1/2 immunoblotting analysis. Quantitative analysis of various embryonic phenotypes (embryo size, weight, digit length) also revealed a tight relationship between the extent of development of embryos with different combinations of *Erk1* and *Erk2* alleles and total ERK1/2 activity in embryonic tissues. As a second approach, we asked whether ERK1 can substitute for ERK2 in mouse embryonic development. We found that ubiquitous expression of an *Erk1* transgene fully rescues the placental and embryonic defects observed in ERK2-deficient embryos. ERK1-only mice grow normally, are fertile and do not display any overt phenotype. Expression of transgenic ERK1 also rescued the proliferation defect of ERK2-deficient MEFs and restored normal phosphorylation of a panel of ERK1/2 substrates. Our study provides compelling and definitive evidence for a functionally redundant role of ERK1 and ERK2 kinases during development (Fremin et al., 2015). Interestingly, Aiodi et al. recently reported a similar functional redundancy of the upstream kinases MEK1 and MEK2. Knock-in of *Mek2* at the *Mek1* locus rescued the placental phenotype of MEK1-deficient mice (Aoidi et al., 2016). These observations reinforce the notion that MEK1 and MEK2 isoforms activate ERK1 and ERK2 indiscriminately.

Differences in the phenotypes of *Erk1* and *Erk2* null mice are attributable to differences in expression levels, with ERK2 being the predominant isoform. The higher expression level of *Erk2* in most mammalian tissues can be related to a stronger promoter, although further regulation by post-transcriptional mechanisms cannot be ruled out (Busca et al., 2015). Importantly, ERK1 and ERK2 proteins have a long half-life of over 50 h (Schwanhausser et al., 2011) and no evidence of stimulus-induced change in protein levels has been reported, indicating that ERK1/2 protein expression levels are not subject to regulation by feedback mechanisms. Furthermore, genetic disruption of a single ERK isoform does not result in increased expression of the other isoform as documented by immunoblotting analyses (Saba-El-Leil et al., 2003; Voisin et al., 2010; Fremin et al., 2015).

**Figure 1:**
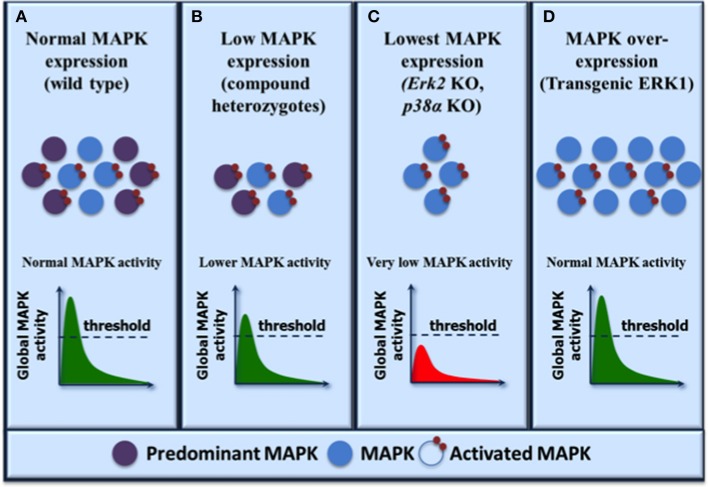
**Functional redundancy within the MAP kinase family: a model to reconcile biochemical and genetic evidence**. The expression levels of MAP kinase isoforms are shown on the top and the resulting activation levels are illustrated graphically as global MAP kinase activity. A threshold of activity is required for normal biological output. MAP kinase isoforms are activated by upstream MAP kinase kinases indiscriminately and the resulting global MAP kinase activity depends on the level of expression of the individual isoforms. **(A)** In normal physiological conditions, both MAP kinase isoforms are activated with the predominantly expressed kinase contributing to most activity. **(B)** Reduced MAP kinase expression results in decreased global MAP kinase activity but the activity remains above threshold resulting in normal phenotypic outcome as exemplified by compound heterozygotes (ERK1/2, JNK1/2, or p38α/β). **(C)** Further reduction of MAP kinase expression (by depletion of the predominant kinase isoform) lowers global MAP kinase kinase activity below the threshold and results in developmental defects (ERK2 or p38α) or deficient cell proliferation (ERK2, JNK1). **(D)** Overexpression of the less predominant kinase restores global MAP kinase activity above threshold and rescues the phenotypes associated with the loss of the predominant kinase (transgenic ERK1). Note that in this model, the maximum level of MAP kinase activity is dictated by upstream activators such that the same global activity is observed under normal or MAP kinase overexpression conditions.

The above findings underscore the concept that a threshold of global ERK1/2 activity determines developmental progression and phenotypic outcome (Figure 1). ERK1 and ERK2 provide a pool of functionally interchangeable kinases available for activation and different thresholds of ERK1/2 activity are required for executing different developmental decisions in specific cellular contexts. In most cells or tissues where ERK2 is the predominant isoform, loss of ERK2 results in a greater decrease of total ERK1/2 activity associated with a broader spectrum of phenotypic manifestations as observed in many studies. Overexpression of the non-predominant ERK1 isoform is sufficient to replenish the pool of ERK kinases, restore global ERK1/2 activity and rescue the ERK2-associated defects. Thus, phenotypes resulting from the depletion or genetic deficiency of a single MAP kinase isoform should be cautiously interpreted in the context of global MAP kinase activity.

The group of Philippe Lenormand recently reported the most detailed analysis of the expression and evolution of ERK1 and ERK2 protein sequences in vertebrates (Busca et al., 2015). Interestingly, they found that the *Erk1* gene has been lost in all bird lineages and some amphibians, whereas squamates only express ERK1 isoform, despite the presence of both *Erk1* and *Erk2* genes. The finding that tetrapods can live by expressing only ERK1 or ERK2 provides further demonstration of the functional redundancy of ERK isoforms in animal physiology.

## Redundancy of MAP kinases: The case of JNK1 and JNK2

The JNK pathway provides another example where different groups have reported contradictory conclusions about the specificity or redundancy of closely related MAP kinase isoforms. JNK1 and JNK2 are ubiquitously expressed in the mouse although their expression levels vary across tissue types. Mice deficient in either *Jnk1* or *Jnk2* gene exhibit distinct phenotypes, suggesting that individual JNK isoforms may serve different signaling functions (Davis, 2000). In addition, *Jnk1*^−∕−^ and *Jnk2*^−∕−^ MEFs proliferate at different rates, a phenotype that has been related to the expression levels of *cJun* (Tournier et al., 2000; Sabapathy et al., 2004). Specifically, JNK1-deficient MEFs have lower cJun levels and proliferate more slowly than wild type MEFs as opposed to JNK2-deficient MEFs that express higher levels of cJun and proliferate faster. These findings have led to the hypothesis that JNK1 and JNK2 have distinct and opposite roles in regulating cJun expression and cell proliferation (Ronai, 2004; Sabapathy and Wagner, 2004).

The group of Roger Davis has revisited the proposed negative regulatory role of JNK2 on cJun expression and cell proliferation using a chemical genetic approach (Jaeschke et al., 2006). They introduced the M108G mutation in JNK2 to enlarge the ATP binding pocket and render the kinase sensitive to the small molecule inhibitor 1-NM-PP1 (Bishop and Shokat, 1999). The mutant *Jnk2*^*M108G*^ allele was integrated at the endogenous *Jnk2* locus by homologous recombination to generate mice expressing analog-sensitive JNK2 kinase. Analysis of MEFs bearing different combination of *Jnk1* and *Jnk2* alleles revealed that pharmacological inhibition of JNK2 in JNK1 proficient cells caused no change in cJun expression or cell proliferation, contrary to the results obtained in *Jnk2*^−∕−^ cells. However, both genetic ablation and pharmacological inhibition of JNK2 in *Jnk1*^−∕−^ cells reduced cJun levels and inhibited cell proliferation. These results demonstrated that JNK1 and JNK2 act redundantly to increase cJun expression and promote cell proliferation. The most likely explanation for the misleading phenotype of *Jnk2*^−∕−^ cells is that loss of JNK2 leads to increased JNK1 function by a compensatory adaptation mechanism. This adaptation is not observed upon acute inhibition of the kinase and/or in conditions where protein expression is maintained. This study also highlighted the importance of using multiple experimental approaches to interpret the phenotypes of mouse mutants, as discussed above for ERK1 and ERK2.

## p38α/β and p38γ/δ kinases have overlapping roles

The p38 MAP kinase pathway regulates numerous cellular processes including adaptation to environmental stress, innate immunity; cell cycle progression and cellular differentiation (Cuenda and Rousseau, 2007; Cuadrado and Nebreda, 2010; Trempolec et al., 2013). The mammalian p38 kinase family is composed of four members, p38α, p38β, p38γ, and p38δ, of which the p38α and p38β isoforms are the closest related isoforms with 75% amino acid identity (Cuenda and Rousseau, 2007). The two isoforms are ubiquitously expressed, although p38α is the predominant isoform in most tissues. p38α and p38β are commonly activated by a wide variety of environmental stresses or inflammatory cytokines and share similar substrate specificity, suggesting overlapping functions. Gene disruption studies have revealed that *p38*α and *p38*β mouse mutants exhibit distinct phenotypes. Specifically, loss of p38α is embryonic lethal owing to defects in placenta morphogenesis (Adams et al., 2000; Allen et al., 2000; Mudgett et al., 2000; Tamura et al., 2000). Conditional deletion of p38α in the mouse embryo bypasses the embryonic lethality but mice die shortly after birth as a result of lung dysfunction (Hui et al., 2007). In contrast, the p38β knockout is viable with no obvious phenotype (Beardmore et al., 2005). This suggests, but does not prove, that the two kinases may have specific roles in certain tissue types.

The group of Angel Nebreda used a combination of genetic approaches to address the question of the specificity and redundancy of p38α and p38β isoforms (del Barco Barrantes et al., 2011). Their work suggested a specific role for p38α in placental development since the placental defects resulting from p38α deficiency could not be rescued by expression of a p38β knock-in allele under transcriptional control of the endogenous p38α promoter. On the other hand, several embryonic phenotypes including defects in heart development, spina bifida, and exencephaly were observed in compound *p38*α and *p38*β deficient embryos but were absent in single gene knockouts, indicating that that the two isoforms can compensate for each other with respect to these defects (del Barco Barrantes et al., 2011). These results demonstrate that p38α and p38β have overlapping functions suggesting functional redundancy of the two MAP kinase isoforms. Consistent with this idea, the phenotypes observed were found to be dependent on the dosage of p38α and p38β. Specifically, embryos with a single *p38*β knock-in allele in the p38 knockout background developed to E18.5 and showed rescue of spina bifida and exencephaly defects, but not heart defects. Upon increased dosage from the two additional endogenous *p38*β alleles, the heart anomalies were rescued and, more importantly, some of the animals survived to adulthood, thereby overcoming the lung defects observed in p38α-deficient animals (del Barco Barrantes et al., 2011). These results are reminiscent of our observations in which specific tissues requiring high levels of global ERK1/2 activity showed defective development in absence of the predominantly expressed ERK2 isoform, which could be completely rescued by overexpression of ERK1 thus confirming that the two kinases are interchangeable and that gene dosage is crucial (Fremin et al., 2015). In the case of p38 isoforms, the possibility also exists that the placental defects that persist in p38β knock-in mice are simply the consequence of insufficient p38β expression. The use of a transgenic approach in which higher ubiquitous expression levels of p38β can be achieved may rescue this phenotype in which case this would demonstrate that the two kinases are redundant.

Further evidence that p38α and p38β act redundantly comes from work demonstrating that embryos lacking both *p38*α and *p38*β genes are deficient in sex determination due to reduced expression of the testis-determining gene *Sry* (Warr et al., 2012). In another study, compound loss of p38α and p38β was shown to compromise Met signaling to p53 in the developing liver. The loss of p53 Ser 389 phosphorylation by p38 MAP kinases in mutant livers resulted in increased hepatocyte death (Furlan et al., 2012). In these two studies, the phenotypes associated with the loss of p38α and p38β were absent in mice deficient in one of the two isoforms.

The two other members of the p38 MAP kinase subfamily also exhibit overlapping functions. p38γ and p38δ share 70% amino acid identity (Cuenda and Rousseau, 2007) and mice deficient for a single p38γ or p38δ isoform show no obvious phenotype under normal physiological conditions (Sabio et al., 2005, 2010; Remy et al., 2010; Risco and Cuenda, 2012). However, disruption of both *p38*γ and *p38*δ genes has unveiled key roles of p38γ and p38δ isoforms in tissue regeneration, innate immune responses, inflammation, and tumorigenesis (Escós et al., 2016). Analysis of compound p38γ and p38δ deficient mice revealed that both kinases are required for physiological and pathological cardiac hypertrophy (Gonzalez-Teran et al., 2016). The role of p38γ and p38δ in the inflammatory response was documented in various experimental mouse models such as lipopolysaccharide-induced septic shock and acute liver failure (Risco et al., 2012; Gonzalez-Teran et al., 2013) and collagen-induced arthritis models (Criado et al., 2014). Similarly, in models of cancer associated with chronic inflammation, such as colitis-associated cancer (Del Reino et al., 2014) and the two-step chemical skin carcinogenesis model (Zur et al., 2015), deficiency in p38γ and p38δ was shown to decrease cytokine production and immune cell infiltration, resulting in decreased tumor burden. Together, these studies suggest that p38γ and p38δ isoforms may exert functionally redundant roles. More rigorous genetic approaches similar to those used to demonstrate functional redundancy of the ERK1/2, JNK1/2, or p38α/β should be used to formally address this question for p38γ and p38δ.

## Concluding remarks

We have used a combination of genetic approaches together with quantitative analysis of embryonic phenotypes and ERK1/ERK2 activity to demonstrate that ERK1 and ERK2 isoforms are functionally redundant in mouse development and physiology. This conclusion is consistent with the discovery of animal species that express only ERK1 or ERK2 and with studies showing that the two isoforms share similar biochemical properties and substrate specificity. Similarly, by combining multiple experimental approaches, other studies have revealed that JNK and p38 MAP kinase isoforms exert functionally redundant roles. These findings clearly illustrate the importance of using multiple genetic, pharmacological and phylogenetic analyses to define the physiological functions of related signaling proteins.

The question of MAP kinases redundancy has far-reaching implications. Dysregulation of the ERK1/2, JNK1/2/3, and p38α/β/γ/δ pathways has been causally linked to human congenital syndromes and to a variety of diseases including cancer, arthritis, fibrosis, cardiomyopathies, and neurodegenerative diseases. Small molecule inhibitors of the ERK1/2 pathway have been approved for the treatment of BRAF^V600E^ metastatic melanoma and other inhibitors of MAP kinase pathways are undergoing clinical evaluation. It is therefore crucial to determine whether the direct or downstream targets of these inhibitors have specific or redundant functions.

## Author contributions

All authors listed, have made substantial, direct, and intellectual contribution to the work, and approved it for publication.

### Conflict of interest statement

The authors declare that the research was conducted in the absence of any commercial or financial relationships that could be construed as a potential conflict of interest.
